# Sexual Health Help-Seeking Behavior among Migrants from Sub-Saharan Africa and South East Asia living in High Income Countries: A Systematic Review

**DOI:** 10.3390/ijerph15071311

**Published:** 2018-06-22

**Authors:** Donna Angelina Rade, Gemma Crawford, Roanna Lobo, Corie Gray, Graham Brown

**Affiliations:** 1 School of Public Health, Curtin University, Kent Street, Bentley, WA 6102, Australia; d.rade@postgrad.curtin.edu.au; 2 Collaboration for Evidence, Research and Impact in Public Health, School of Public Health, Curtin University, Kent Street, Bentley, WA 6102, Australia; g.crawford@curtin.edu.au (G.C.); roanna.lobo@curtin.edu.au (R.L.); Graham.Brown@latrobe.edu.au (G.B.); 3 Australian Research Centre in Sex, Health and Society School of Psychology and Public Health, La Trobe University, Bundoora, VIC 3086, Australia

**Keywords:** migrants, sexual health, help-seeking behavior, systematic review

## Abstract

The number of migrants has increased globally. This phenomenon has contributed to increasing health problems amongst migrants in high-income countries, including vulnerability for HIV acquisition and other sexual health issues. Adaptation processes in destination countries can present difficulties for migrants to seek help from and gain access to health services. This study examined migrants’ from sub-Saharan Africa (SSA) and South East Asia (SEA) sexual health help-seeking behavior in high-income countries with universal health coverage. The systematic review followed PRISMA guidelines and was registered with PROSPERO. Several databases were searched from 2000 to 2017. Of 2824 studies, 15 met the inclusion criteria. These consisted of 12 qualitative and three quantitative studies conducted in Australia, Spain, the United Kingdom, Belgium, Scotland, Ireland, and Sweden. Migrants experienced a range of difficulties accessing health services, specifically those related to sexual health, in high-income countries. Few studies described sources of sexual health help-seeking or facilitators to help-seeking. Barriers to access were numerous, including: stigma, direct and indirect costs, difficulty navigating health systems in destination countries and lack of cultural competency within health services. More culturally secure health services, increased health service literacy and policy support to mitigate costs, will improve health service access for migrants from SSA and SEA. Addressing the structural drivers for stigma and discrimination remains an ongoing and critical challenge.

## 1. Introduction

The number of migrants has been increasing globally, with an estimate of 258 million international migrants in 2017 [1]. Between 1990 and 2017, the number of international migrants increased from 152.5 million to 257.7 million—a rise of over 69% [1]. Half (51%) of these migrants were situated in only 10 countries—nine of which are classified as high-income countries (HIC) by the World Bank, including United States of America (USA), France, Canada and Australia [2]. A documented migrant is defined as someone “who entered a country lawfully and remains in the country in accordance with his or her admission criteria” [3]. Australia, as an example, has a substantial proportion of documented international migrants [4] with the Australian Bureau of Statistics [4] recently reporting that the number of overseas migrants has reached its highest point in 20 years.

There are multiple and complex reasons for migration to HIC, including: safety and security, health, labour, economic inequalities between high and low-income countries, and family reunification [5,6,7]. There are a range of health issues associated with migration; with increased vulnerability during the migration process [8]. Research from HIC indicates migrants from low and middle-income countries (LMIC) may have different health and health care needs than the destination population [8]. This vulnerability may lead to specific problems regarding sexual and reproductive health (SRH) and service provision [9]. The complexity of migrants’ diverse religions, cultural backgrounds, educational levels, migration histories, present living conditions and legal statuses may all influence their sexual health (SH), including their vulnerability for HIV acquisition [9].

Research with migrants in Australia, Canada, the United Kingdom (UK), France, Switzerland and the Netherlands highlight vulnerability for HIV acquisition, with some overseas born populations overrepresented in HIV notifications [10]. In the case of Australia, people born in sub-Saharan Africa and North and Southeast Asia have some of the highest rates of HIV diagnoses by region of birth and are overrepresented in late or advanced presentation of HIV infection [11]. It has been suggested that a range of risk factors have contributed to the higher number of HIV cases from these two regions, such as: low levels of HIV literacy; high rates of undiagnosed and therefore untreated HIV infections; significant levels of stigma attached to HIV which prevents people from being tested, treated, or from seeking information; and misconceptions that Australia is free from HIV [12].

Personal, interpersonal, social life, environmental, and cultural factors play important roles in influencing migrants’ sexual health help-seeking behavior. Previous Australian research shows SEA and SSA face a range of challenges, such as the adaptation process, cultural and gender differences and norms, level of control over decision-making, lack of familiarity with the Australian health system, lack of English language proficiency and inequities in health service access, including to HIV testing [12]. Whilst not explored in this review, it is acknowledged that there are further barriers to accessing health services for undocumented migrants.

A better understanding of the facilitators and barriers faced by documented migrants from SSA and SEA in utilizing SRH services may inform the development of tailored and innovative health promotion strategies [13] and more comprehensive, appropriate, and accessible health services for migrants in high income countries. The aim of this systematic review is to explore, from the perspective of migrants, types of help-seeking behavior and the barriers and enabling factors that influence documented migrants in gaining access to SRH services and HIV testing in high income countries (such as Australia) with universal health coverage (UHC).

## 2. Methods

This systematic review was conducted according to the Preferred Reporting Items for Systematic Reviews and Meta-Analyses (PRISMA) guidelines [14]. It followed procedures previously used by other systematic reviews conducted by the research team [15,16,17]. The review was registered in the International Prospective Register of Systematic Reviews (PROSPERO) to ensure quality adherence, reporting and dissemination (registration number: CRD42015023330).

### 2.1. Search Strategy and Information Sources

A total of seven databases were searched: PsycINFO, MEDLINE, ProQuest, PubMed, Scopus, Global Health and Web of Science. Google Scholar was also used to verify the results of the database searches. Lastly, an examination of reference lists from the relevant studies was undertaken to assess whether database results were exhaustive.

Search terms included: (migrant*, immigra*, “sexual health”, “reproductive health”, “help-seeking behavio?r, “health seeking behavio?r”). All relevant variations, including Medical Subject Headings (MeSH) [18] terms were used depending on database requirements and specifications (see Table 1). Searched fields were keywords, title, and abstract.

This review considered qualitative or quantitative evidence from primary studies related to adult migrant sexual health help-seeking behavior in HIC. For the purposes of this study, HIC were those nominated by the World Bank as Organization for Economic Co-operation and Development (OECD) countries with Gross National Income (GNI) per capita above $12,236 [2]. Only articles with a focus on sexual health were included. Help-seeking behaviour was described as “the behaviour of actively seeking help” and included formal (e.g., from health professionals), informal (e.g., from friends and family) and self-help sources [19].This review was interested in studies of documented migrants, and studies that focused solely on undocumented migrants were not included. This was due to the fact that undocumented migrants experience unique barriers to accessing health services compared to documented migrants. Studies were incorporated if they included migrants from sub-Saharan African and Southeast Asian countries, aged above 18 years old, and residing in high income countries for more than 1 year. This review was interested in the migrant experience, and as such, articles on the perspectives of healthcare providers were not included.

The review included only studies conducted in countries that had Universal Health Coverage (UHC), such as Australia, Spain, the United Kingdom, Belgium, Scotland, the Republic of Ireland, and Sweden. Description of what UHC entails, who is entitled to UHC, and a definition of UHC, has been debated [20] and as such, there are discrepancies in its applications between countries for migrants. This review used as the definition of UHC a “health care system that provides healthcare and financial protection to more than 90% of the citizens of a particular country” [21]. Data from the Global Residence Index was used to determine countries with UHC [21]. From this list, only two HIC were without UHC—the United States of America and Saint Kitts and Nevis. It is acknowledged that UHC is often not extended to all migrants. For example, those on temporary visas (including international students and workers) in Australia are not eligible to Medicare (Australia’s UHC system) [22]. Likewise, for those seeking asylum in Australia, health care is reported to have been below Australian standards, with long delays in accessing medical professionals [23]. Broader articles on help-seeking or reproductive health only were not included.

Only full text, peer-reviewed journal articles, published in English, between the years 2000–2017 were included in the review. Every study identified through the database searches was examined based on information in the abstract and title. The full text from relevant studies that met the inclusion criteria was then retrieved.

**Figure 1:**
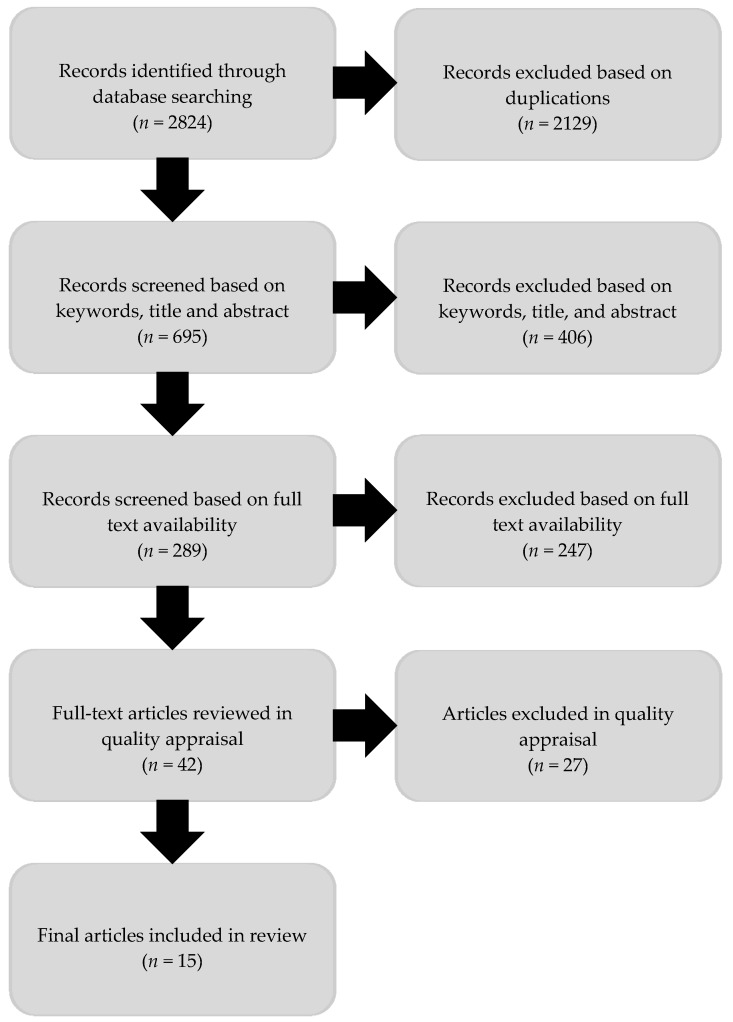
Flow diagram of review process.

Title and abstract were screened by two reviewers for potential eligible articles. The results were then checked by another member of the team based on the inclusion and exclusion criteria. Forty-two articles were eligible. Quality appraisal was conducted by two reviewers using a checklist adapted from the Joanna Briggs series of assessment and review instruments [24] and the National Institute for Health and Care Excellence (NICE) Quality Appraisal Checklist [25]. Criteria considered in the quality appraisal checklist included: study population, methodology, outcomes and analysis. Meetings to discuss quality appraisal with the broader research team identified additional articles for exclusion based on the income of the host country, UHC status and migrant country of birth. Final judgment regarding the quality of selected studies was based on the overall assessment of the article, methods, and study objectives and their relevance. Studies that were excluded at this point (*n* = 27) included those without ethical approval or insufficient content on sexual health and HIV. A final 15 studies were included. Figure 1 shows the process of study selection.

### 2.2. Data Extraction

Two researchers independently performed data extraction on the selected articles using a standardized data extraction form [24]. The data extracted included author and title, research objective, study design, and key conclusions and recommendations. The final studies were classified into three broad areas: (1) Sources of sexual health help-seeking of migrants, (2) enabling factors for sexual health help-seeking behavior of migrants, and (3) barriers to sexual health help-seeking behavior of migrants.

## 3. Results

Fifteen studies met the inclusion criteria for the review (see Table 2). All selected studies included migrants from Southeast Asia and sub-Saharan African countries living in high income countries, specifically those with UHC. Of these 15 studies, seven were conducted in Australia [26,27,28,29,30,31,32]; two in the UK [33,34] and one study in Sweden [35], Ireland [36], Spain [37], Belgium [38], and Scotland respectively [39]. Of the 15 studies, eight studies examined migrants from sub-Saharan African countries [29,31,33,34,36,38,39,40]; two studies examined migrants from Southeast Asia countries [26,35]; and five studies explored migrants from mixed regions [27,28,30,32,37].

Twelve of the studies were qualitative [26,27,30,31,32,33,34,35,36,37,38,40]; and three were quantitative studies [28,29,39]. Of the 12 qualitative studies, six used focus group discussions [26,27,31,33,34,38], three used in-depth interviews [32,35,36,37], and three used a mix of focus group discussions and interviews [30,36,40]. Nine of the studies included both male and female participants [27,28,30,32,33,34,36,38,40], with the remaining six studies completed with female participants [26,29,31,35,37,39]. Three of the studies focused only on refugees [26,29,30], and four of the studies had a mean arrival to destination country of less than five years [27,29,35,36]. The studies varied in participant age with the majority including participants between 18 and 60 years. Two studies included participants aged 16 and over [30,39]. Two of the studies were conducted with people living with HIV [32,37]. All studies received ethics approval from a human research ethics committee [26,27,28,29,30,31,32,33,34,35,36,37,38,39,40].

Results have been reported on in the following domains:

- Sources of help-seeking—sources of information for sexual health and sources for treatment.
- Facilitating factors—social support and patient-healthcare provider relationship.
- Barriers—personal factors; interpersonal and cultural factors; cultural competency of healthcare provider; healthcare cost and location; confidentiality and relationship with healthcare provider.
- Study recommendations—policy, practice (clinical and health promotion) and research.

### 3.1. Sources of Sexual Health Help-Seeking

Of the 15 included studies, four studies reported findings on the sources of sexual health information sought by migrants from sub-Saharan African and Southeast Asian countries living in high-income countries [27,30,33,35,39].

Migrants used a range informational sources including health professionals, TV and radio, books and magazines, and friends and family. Generally, there was a preference for information to come from a professional source; however, this was not easily accessed [30,39]. For example, in a survey with African women in Scotland (*n* = 92), Yakubu et al., found that the majority of women received SH information from books and magazines (79%); TV/radio (76%); and friends and family (70%); and only around a third from a family doctor (35%) [39]. However, preferences on where to receive information were firstly from a doctor (57%), a sexual health clinic (46.6%) and TV/radio (39.7%), with only a small proportion wishing to receive information from family and friends (27%).

A study by McMichael and Gifford used focus groups and interviews to explore access to sources of sexual health information and services among young (16–25 years old) refugees in Australia (*n* = 142). They found a preference for participants to seek sexual health information from doctors, who were seen as having expertise and the ability to provide accurate information [30]. However, it was highlighted that the participants only sought help from a doctor in relation to symptoms of an STI, for contraception or for an unplanned pregnancy [30]. In addition, participants tended to discuss sexual health with their peers, however, they reflected that they could not rely on their friends’ information because it was sometimes unreliable and incomplete. Participants found it difficult to seek SH information from their parents due to religious and cultural factors; fear of judgement; and dislike for responses that focused on warnings or personal responsibility [30].

In this study, media (including radio and TV) and written material (books and magazines) were common sources of information; however, young refugees suggested that some of these materials presented were uninteresting, or provided exaggerated or inconsistent information [30]. Instead, the study found preference for using the internet as a source of sexual health information, as users can remain anonymous [30].

In semi-structured interviews with Thai women in Sweden (*n* = 19), Akerman et al. found that most women would discuss sexual health information with their partner in the first instance [35]. In the case of contraceptives or other women’s health issues (i.e., pregnancy), women would seek information from other women, including Thai friends, family, or their partner’s family [35]. Women also used the internet before visiting their GP (accessed via their partners) [35].

A survey by Drummond et al. with newly arrived (less than 5 years) West African women in Australia (*n* = 51) found that most women would access a medical clinic (94%) or a hospital clinic (67%) if they thought they had a STI [29]. They were least likely to seek treatment from a religious leader (8%), self-help groups, family or friends or traditional healers (all 10%) [29].

### 3.2. Facilitators for Sexual Health Help-Seeking Behavior

Of the 15 studies, five studies described factors that facilitated migrants to access and utilize sexual health services [27,28,30,35,37]; however, these were often not the main research objective.

In some studies, participants described attending healthcare services if there was a physical symptom that they could not fix [30,35]. Social support was not only a source of information on SRH, but also supported help-seeking behavior [27,28,35,37]. In semi-structured interviews by Guionnet et al. with women living with HIV in Spain (*n* = 26), participants reflected that having the support of loved ones assisted them to adhere to treatment, process the information from their doctor and encouraged them to continue their treatment [37]. Likewise, positive relationships with their healthcare provider encouraged participants to attend appointments and adhere to treatment. Positive interaction included: the doctor being an expert on the issue, providing emotional support and translation of scientific terms to lay language [37]. A trustworthy relationship with healthcare providers was also found to facilitate access to sexual health services [30,37].

### 3.3. Barriers to Sexual Health Help-Seeking Behavior

Fourteen of the 15 studies highlighted factors that inhibited migrants from accessing health services in high income countries [27,28,29,30,31,32,33,34,35,36,38,40]. From these studies, the following barriers to accessing health care services were described.

#### 3.3.1. Personal Factors

Studies indicated that many migrants from sub-Saharan African and Southeast Asian countries had inadequate information about the health system in destination countries and experienced difficulty navigating access, such as how to make appointments or knowing the necessary documents required [27,32,34,35,40]. Poor knowledge and understanding of primary healthcare also limited migrants’ access [30,38]. In addition, many studies reported a lack of knowledge of available SRH services and their location [27,33,35,38].

#### 3.3.2. Interpersonal and Cultural Factors

Eight of the 15 studies examined interpersonal factors preventing migrants from seeking help from health services [26,27,29,30,33,34,36,38]. Barriers cited by study participants were: feeling afraid of being judged by the health provider; feeling afraid and ashamed about what other people would think; and feeling embarrassed to discuss sexual health [26,27,29,30,36,40]. In a study by Drummond et al., participants who were asked about accessing services for an STI expressed concern about losing their job, being afraid of treatment and medication used, and were of the belief they could cope with the problem themselves [29].

In focus groups (*n* = 70 participants) by Manirankunda et al., sub-Saharan African migrants living in Belgium expressed HIV-related stigma, guilt and shame, which included a belief that people living with HIV were “*at fault*” for their diagnosis [38]. This type of stigma resulted in participants feeling that they were not at risk (as they had not engaged in ‘bad’ behavior) or avoiding testing due to fear of being diagnosed [38]. Additionally, in focus groups and interviews (*n* = 60 participants) by Adedimeji et al., sub-Saharan African participants living in Ireland expressed concern about the perception that HIV is only for “*people from Africa*”, which created feelings of mistrust and suspicion among their own migrant communities [36]. Participants also reflected that HIV stigma was an issue, particularly among religious and community leaders. This resulted in gossip surrounding the HIV diagnosis of an individual, and in the individual feeling isolated and unsupported by their community. These experiences had a negative impact on people’s willingness to test for HIV [36].

Likewise, there was a perception among participants in a number of studies that a sexual health issue (such as a pregnancy outside of marriage or a HIV diagnosis) would result in social isolation [26,38,40]. Guionnet et al. found that women living with HIV did not wish to disclose their status due to fear of being socially rejected, fear of being labelled as ‘positive’ or associated with negative stereotypes, and fear of hurting loved ones [37]. All women who did not disclose their status to a loved one stopped treatment [37]. In focus groups and interviews by Lindkvist with migrants from Ethiopia and Eritrea living in Sweden (*n* = 28), fear of being known to be living with HIV resulted in people not telling loved ones about their diagnosis and travelling to other cities for testing and treatment [40].

In a quantitative study using Q methodology (*n* = 42), Dune et al. found that for migrants living in Australia, perception by others influenced help-seeking behavior [28]. Lack of support for issues such as STIs and having sex outside of marriage made people reluctant to seek professional care or seek appropriate resources (i.e., condoms) [28,30,37,40]. Focus groups by Roger et al. with Sudanese and Eritrean mothers and daughters in Australia highlighted a lack of discussion around SRH topics in their communities, with sexual health considered taboo [31]. As a result, many women were hesitant to discuss sexual health with a healthcare provider and had difficultly using the correct terms for SRH [31]. This finding was similar across a number of other studies [26,27,30].

In focus groups with refugee women from Karen (*n* = 42) living in Australia, Ussher et al. found conflict between refugees’ culture and beliefs and Australia’s approach to sexual education [26]. While older women expressed concern that teaching young girls about sexual health would encourage sex outside marriage, they found it difficult to control access to movies featuring representations of sex, sexual health education at school and interaction with sexually active friends. For young women, the taboo nature of sexual health made them reluctant to seek information or access services regarding their sexual health.

For women from Thailand in the Åkerman et al. study, access to health services was delayed or avoided due to perceived dependence on their male partner [35]. The male partner was first consulted, before he would then arrange for his female partner to attend a health service. Likewise, in interviews with migrants living with HIV in Australia (*n* = 29) in a study by Korner et al., women also indicated dependence on their husband to arrange healthcare appointments [32].

In some studies, participants preferred the use of traditional medicines—either entirely, or in conjunction with prescribed medication [33,34,38]. Participants also reported self-medicating until they were “really” sick, before seeking professional treatment [34].

#### 3.3.3. Cultural Competency of Healthcare Provider

Cultural competency issues were reported as barriers in eight studies [27,31,32,33,35,36,37]. As an example, in a study by Adedimeji et al., sub-Saharan African migrants living in Ireland expressed reluctance to seek healthcare due to perceived poor service from providers; with the perception that health providers were insensitive and arrogant [36]. This was instigated by a lack of appropriate interpreter services and a perception that providers believed “*everyone from Africa is living with HIV*”, resulting in perceived discrimination [36]. This was also a concern expressed in focus groups by Agu et al., with migrants from SSA and SEA in Australia (*n* = 45), with some participants believing they had been tested without giving consent [27]. Testing without consent was also the experience for some migrants diagnosed with HIV in Australia in the Korner et al. study, who indicated that they were not aware they had been tested when they found out their HIV diagnosis [32]. Experiences of discrimination in the destination country outside of healthcare services also limited willingness to seek professional services [27,33].

The cultural background of a service provider was an important factor in accessing health services [31,33]. In a study by Shangase and Egbe, focus group discussions with Africans living in the UK (*n* = 30) found that participants perceived a “*negative attitude*” from healthcare providers based on their ethnicity [33]. Migrants in some studies suggested that people outside their own culture would be unable to understand their specific needs and would not be in a position to help navigate them through the differences in culture [31,33]. Dune et al. found that participants reported a difference in the culture of their country of origin to Australia in relation to SRH. The study highlighted a belief among participants that healthcare providers were not equipped to deal with their unique needs [28]. Several studies also described the importance of the gender of the healthcare provider, with many women unwilling to discuss sexual health with a male [31,33].

Additionally, a number of studies identified that language was a barrier [30,31,32,33,35,37,40], in that either a provider would not use simple descriptions, or an appropriate interpreter was not available or offered. This resulted in confusion around diagnosis and treatment, including correct dosage [33]. In the Åkerman et al. study with Thai women living in Sweden, male partners were often their interpreter, although they also struggled with the language used in consultations [35]. Participants in this study also expressed a preference to seek healthcare in their country of origin, due to the barriers experienced in accessing their destination country’s health system [35].

In a study by Lindkvist with migrants from Ethiopia and Eritrea living in Sweden, some participants described the misuse of interpreters [40]. Participants described being provided an interpreter without being consulted on whether they needed one which was experienced as an insult. Issues arose when interpreters had conflicting translations, or provided incomplete translation, which created further confusion [40].

#### 3.3.4. Healthcare Cost and Location

Five of the 15 studies found that the cost of healthcare and location were factors that inhibited migrants from accessing health services [27,29,34,36,38].

Adedimeji et al. reported that the cost for GP-provided HIV testing, approximately 40–50 Euros, was the largest barrier to testing for participants [36]. Many participants self-identified as having a low-income, but were not eligible for a medical card (for low-cost health services) [36]. For those able to access government funded health services, there were long waiting times, which resulted in missed opportunities for early diagnosis and linkages to care [36]. Focus groups with sub-Saharan African migrants (*n* = 70) in England by Thomas et al., identified instances of individuals using false identities in order to access free health services [34].

Several studies also found that beyond the cost of consultation, there were other costs experienced by migrants in accessing healthcare [34,36]. These included contraception, pharmaceuticals, time off work, childcare and transport [36,37,38]. In the study by Thomas et al., participants reported importing medication from their country of origin due to the cheaper price [34]. Likewise, in a study by Korner et al., temporary residents living with HIV in Australia indicated they were unable to afford HIV treatment and instead imported treatment from their country of origin [32].

Location of health services was also highlighted as a factor inhibiting access to testing [36,37,40]. Adedimeji et al. reported that most participants were accustomed to seeking treatment from main hospitals, and were unaware there were several private HIV testing services in the capital city of Ireland (Dublin) [36]. The respondents of the study were opposed to accessing treatment in the hospitals due to perceived lack of privacy [36].

#### 3.3.5. Confidentiality and Relationship with Healthcare Provider

Seven of 15 studies addressed the healthcare provider and patient relationship [27,28,30,32,33,34,36].

In a number of studies, participants expressed concern about the confidentiality of their consultation [27,30,31,36,37,40], with some preferring a person outside the community to conduct testing. Additionally, participants in some studies expressed concern about an HIV diagnosis reported to the government or immigration authorities, which they perceived would result in them being deported [27,32,34,36,38].

Negative relationships and previous negative experiences with healthcare providers also limited uptake of health services and testing [37].

### 3.4. Study Recommendations

Fourteen of the 15 studies made recommendations relating to policy, health promotion and clinical practice and research. Recommendations generally related to improvements in clinical practice or for interventions to increase education and access to services [27,29,30,31,32,33,36,37,39,41], such as outreach HIV testing, increased sexual health education, addressing HIV-related stigma, or cultural competency training of clinical staff. Recommendations for policy are generally related to cost of health services and promoting free services [34,35,40], or development of migration policies for people living with HIV [32]. Recommendations for research centered on the need for better understanding of the construction of health and sexual health among migrants and additional barriers to accessing sexual health services [26,28].

## 4. Discussion

There is an upward trend in the number of migrants moving to high income countries [42]. As a result, the number of health issues experienced by SSA and SEA migrants living in those countries has increased, including increases in STIs, HIV and other SRH problems. In many countries, there is concern about delayed access for SRH issues, particularly in relation to late diagnosis of HIV [43,44,45]. Despite these concerns, there are limited peer-reviewed studies assessing SSA and SEA migrant sexual health help seeking behavior in high income countries. This review explored sources of information on sexual health and the barriers and facilitators influencing SSA and SEA migrants to gain access to SH services and testing in high income countries. The review identified 15 peer-reviewed articles that met the inclusion criteria published between 2000 and 2017. The articles selected identified a range of barriers, and to a lesser extent, facilitators and sources of help-seeking across a number of levels.

### 4.1. Overview of Findings

Few articles described sources of help-seeking in this review. For most migrants, healthcare providers were often considered to be the preference for information on SRH [27,30,39]. However, issues relating to access to health services, or a reluctance to discuss sexual health topics, resulted in limited knowledge transfer from these ‘trusted’ sources [30,39]. Combined with low levels of access to trusted sources, several studies identified topics of sexual health as being taboo—particularly for young people who were unmarried [26,27,31]. This contributed to low levels of knowledge on sexual health (mainly on safe sex practices) [46]. Indeed, previous research in Australia has identified lower knowledge on a broad range of sexual health topics among SSA and SEA migrants [47,48,49,50,51]. Knowledge regarding transmission and the process of testing for HIV has been shown to increase rates of testing [52,53].

This review identified very few enabling factors to accessing SRH services for SSA and SEA migrants. For most studies, symptoms relating to an SRH issue were most often the motivator for seeking professional help, which is problematic in regards to STIs and BBVs that can be asymptomatic for a period of time, such as HIV. This is consistent with previous research which has identified a reluctance to seek professional help unless physical symptoms are present, for both sexual health issues and other physical and mental health issues [29,44,52,54].

Barriers to sexual health help seeking were commonly reported and were mostly consistent within this review. The main barriers identified included: low knowledge of healthcare system in the destination country and where to access SRH services; the taboo nature of SRH within communities and lack of perceived social support; lack of cultural competency within the healthcare system; the cost and location of healthcare and concerns of confidentiality [27,28,29,30,31,32,33,34,35,36,38].

In some studies, perceived shame and stigma relating to HIV and STIs limited uptake of health services, even when symptoms were apparent. Expectations of community isolation for issues such as HIV, STIs and unplanned pregnancy contributed to low levels of testing and interactions with healthcare providers [29,31]. In the case of HIV, there was a perception that HIV only happened to ‘bad’ people, which lowered individual perception of risk [55]. Previous studies have identified the important role that addressing stigma and increasing social support has in improving knowledge, safe sex behavior and improving sexual health service uptake [44,52,56,57,58].

For the most part, studies in this review identified a lack of responsiveness within health systems to address the needs of migrants as well as low levels of cultural competency among healthcare providers. Difficulties navigating the healthcare system, booking appointments and knowing how to link into health services were described in this review. Pathways to care are often quite different between origin and destination countries and migrants may bring expectations of health services to their new country [59,60]. The perceived culture regarding SRH between origin and destination country are often conflicted for migrants, with ‘Western’ countries suggested to be generally more liberal in their approach [28,59]. In some cases, a perceived invisibility of community within healthcare services limited uptake. Non-community members were perceived as being unable to help navigate cultural differences, creating difficulty in accessing services [33]. For some sub-Saharan African migrants, this was evidenced by preferences for traditional medication and expressed mistrust of ‘Western’ medicine [33]. In previous studies, general practitioners have identified gaps in their own knowledge and understanding of culture, which have negatively impacted on their delivery of care [61]. There is a need for health systems and providers to be able to understand and engage with cultural beliefs and practices, to better address the SH of migrants in their destination country [59,62].

In some studies, migrants perceived healthcare providers to be discriminatory or unable to address language barriers [30,31,32,33,35,37,40]. Sub-Saharan African migrants reported feeling ‘targeted’ by healthcare providers in relation to HIV, who were perceived as assuming “*everyone from Africa is living with HIV*” [36] and incidences of lack of consent to test for HIV were reported [27,32]. Additionally, where language was a barrier, studies identified the lack of interpreter services, and partners or other family members being used instead. In many cases, healthcare providers were criticized for using overly complicated or scientific terms, rather than lay language [30,31,32,33,35,37,40]. Previous research has identified healthcare providers as being integral to facilitating access to health services, and encouraging BBV and STI testing [62]. It is therefore critical that healthcare providers have the cultural competency to build positive, trusting relationships with migrant patients and have adequate resources (such as interpreters or bicultural workers) to support engagement and perceived cultural security [60,61,63].

### 4.2. Implications for Health Promotion, Clinical Practice, Policy and Research

This review highlighted a number of considerations for practice, policy and research. These implications are based on the results and recommendations made in the included studies, unless otherwise stated. These implications are broad, acknowledging that sub-Saharan Africa and Southeast Asian migrants are not homogenous and have a range of experiences that influence barriers and facilitators to help seeking. As recommended by a number of articles from this review, community involvement is critical in the uptake of strategies to improve access to sexual health services to address unique barriers [64,65].

#### 4.2.1. Implications for Research

Studies discussed the differences in cultural beliefs and practices regarding health, preventative health and sexual health. A better working knowledge of communities’ understanding of health and health behaviors is needed to tailor health services and health promotion interventions [44,66]. Future research should consider segmentation of migrant groups by country of birth, gender, age and other relevant factors to better identify and address specific issues for priority groups.

Many of the studies described barriers to health services, with very little discussion on the sources of or facilitators to help-seeking. Better understanding of what works and why is needed, with a focus on successful interventions in health promotion, service delivery and policy changes [36]. Research that focuses on the pathways of care for migrants in seeking help for sexual health issues will provide better working knowledge of experiences of testing and treatment [67].

#### 4.2.2. Implications for Health Promotion Practice

A number of studies made recommendations regarding sexual education, including safe sex practices. All these studies stressed the importance of culturally-appropriate material and delivery of information [27,36]. Indeed, there is need for health promotion interventions to consider the role of culture, sexuality, resettlement process, understanding of preventative health and gender norms in sexual health help-seeking [35,44,60,66]. Previous research with healthcare providers have also acknowledged the need for resources to extend beyond language, and to also consider cultural factors [68]. Points of education recommended included: school-based education [30], during the settlement process [31,35], and social and ethnic media and community events [58].

The role of community and social support was acknowledged in some studies [36]. Studies also highlighted a need for direct involvement of affected communities in the development of targeted prevention and testing programs [39]. Recommendations included involving community and religious leaders in delivery of sexual education and in addressing stigma and judgment associated with unplanned pregnancy, STIs and HIV within communities. Additionally, developing partnerships between communities, healthcare services and health promotion organisations may help develop trust and facilitate access to relevant services by migrants [31]. Diversifying opportunities to access sexual health services may also increase help-seeking behavior [69]. Community-based testing for HIV, STI and hepatitis B have been successful [70], particularly in conjunction with information sessions from healthcare providers [38].

#### 4.2.3. Implications for Clinical Practice

Access to health services and cultural competency of services were the most cited barriers to help-seeking. As global migration continues to increase, there is a need for countries and health services to be more responsive to the needs of their culturally diverse populations. This should be considered in planning health services to ensure that pathways of care are clear and accessible, there is a diversity of staff (both ethnic group and gender), appropriate interpreters and bilingual workers are accessible when required, and staff have cultural competency training [27,33,60,63,66]. Sufficient time must also be available to explain confidentiality and privacy, health concepts, procedures and treatment options fully, and to listen to and discuss concerns regarding cultural practices and beliefs [33,36].

While not a focus of this review, other studies have cited low knowledge of priority populations for specific sexual health issues, such as HIV and hepatitis B, among healthcare providers [61,67]. Additional training, or systems to prompt sexual health discussions, may be a consideration [41]. However, identifying high risk populations may result in communities feeling ‘targeted’, or reify existing differences [36]. Accordingly, strategies to minimize this need to be considered with relevant communities.

#### 4.2.4. Implications for Policy

Cost and location were frequent barriers to accessing health services. Consequently, direct and indirect (i.e., transport, time off work, childcare etc.) costs of access need to be considered by governments in planning the delivery of health services [36]. Experiences of migrants not being eligible for low-cost health service within countries with universal health coverage, particularly in the case of the UK [34], questions whether UHC is accessible for all people within a country [20]. In other cases, where migrants are able to access UHC, issues may pertain to the location of free health services, difficultly in applying for low-cost health care, differences in gender norms in relation to sexual behavior and help-seeking decision making, experiences of stigma and discrimination or lack of clear pathways of care [44,60,66]. Relevant issues need to be identified for specific populations of migrants to encourage uptake of services.

In many of these studies, documented migrants expressed concern regarding deportation, particularly in relation to HIV. Implications of an HIV diagnosis need to be made clearer in cases of mandatory testing (such as in Australia) [27], to avoid creating additional barriers to healthcare access [32].

### 4.3. Study Design and Reporting Strengths and Limitations

Most studies indicated methodological limitations of the research. Limitations that were frequently reported in included studies were: lack of interpreter or translated material and subsequent exclusion of those who did not speak the destination country language/s; convenience sampling (participants were often those connected to a non-government organisation working in sexual health) and social desirability bias. In some instances, studies provided little detail about country of birth, age range and other sociodemographic details, as well as legal status in host country. Most studies recruited participants from multiple countries of birth, had large age ranges and included both male and female participants, with subsequent reporting of very broad, common issues and experiences. Very few studies provided more specific detail for particular groups. Poor understanding of variations between cultures can result in incorrect assumptions about migrant health needs and subsequent low health service utilisation. Positively, all included studies sought ethical approval.

The majority of studies (*n* = 12) reported were qualitative. As such, the association between barriers and facilitators and help-seeking outcomes were not able to be assessed. However, the qualitative nature of these studies does provide further insight into the facilitators and barriers and provided greater detail and context regarding migrants’ experiences. Most studies provided detail about the methodological and theoretical framework and analysis process; however, some were limited in their reporting against best-practice reporting criteria.

Quantitative studies acknowledged the limitation of small sample sizes, with all studies recruiting less than 100 participants, and two studies less than 50. These studies focused on constructs and attitudes towards sexual health help-seeking but did not report on behavior or other help-seeking outcomes.

### 4.4. Strengths and Limitations of the Systematic Review

This systematic review is the first known study to assess sub-Saharan African and Southeast Asian migrants’ sexual health help seeking in high-income countries in relation to sources of help-seeking, facilitators and barriers. The use of seven databases and multiple search terms across 17 years of peer-review literature provided a broad scope of studies. Inclusion of both quantitative and qualitative studies provided both descriptive data alongside participants’ lived experiences and the context of these experiences. Multiple researchers reviewed database search results and assessed the quality of the studies for inclusion to reduce error. The review was registered with the PROSPERO International Prospective Register of Systematic Reviews. While the studies had different research questions, many of the barriers described in this review were consistent.

The limitations of this systematic review were that it included only peer-reviewed studies and those published in English. The exclusion of grey literature in this review may have limited the results, which has implications for external validity. Additionally, only articles published in English were included due to limited resources, which may have narrowed the scope of this review. It is likely that studies in other languages may have provided valuable contributions. No meta-analysis was conducted due to the heterogeneity of the included studies.

The SR only focused on high-income countries that have a universal health care system, therefore the results may not be transferable to countries such as the USA. The selected studies focused only on migrants with legal status in their destination country and there may be additional barriers for undocumented migrants. Future reviews could look at the barriers specific to undocumented migrants, as these are unique factors not relevant to documented migrants. The results of this review were context specific and are unlikely to be transferable to all sub-Saharan African and Southeast Asian migrant populations residing in high-income countries, though the findings may be valuable for a number of countries and provide broad insights and recommendations for research, policy and practice. Many of the studies included focused on barriers to health service access, making it difficult to comment on sources of help-seeking and facilitators.

This review was only interested in migrants’ perspectives. It is acknowledged that studies on healthcare provider perspectives contain a rich range of information, which is not described here. Further work is planned to review this.

## 5. Conclusions

Growth in global migration has seen increases in the acquisition of STIs and BBVs amongst migrants. For migrants travelling to high-income countries from countries in regions of higher HIV prevalence, a range of factors may increase vulnerability for HIV acquisition. This study found barriers to access included: stigma relating to STIs and BBVs, direct and indirect cost associated with access, difficulty navigating health systems in destination country and lack of cultural competency within health services. Very few studies described sources of sexual health help-seeking or facilitators to help-seeking.

Early diagnosis of STIs and BBVs is crucial to prevent onwards transmission and reduce the burden of associated healthcare. Accordingly, a better understanding is required of the structural drivers of inequality including culture and gender along with targeted, resourced and evidence-informed strategies to address these barriers.

## Figures and Tables

**Table 1:** Search terms and databases used in the systematic review.

Databases	PsycINFO, MEDLINE, ProQuest, PubMed, Scopus, Global Health and Web of Science.
Concept 1: Migrants	“Ethnic group*” OR “Culturally and Linguistically Diverse” OR “Non-English speaking” OR “Ethnic minority*” OR “Transient*” OR “migra*” OR “Immigra*” OR “International student*” OR “Migrant worker*” OR “Labour migra*” OR “Minority group*” OR “Asylum seeker” OR “Displaced people”
Concept 2: Sexual health	“Sexual behavio*” OR “Sexual risk behavio*” OR “Sexual practice*” OR “HIV infection*” OR “Sexually transmitted disease*” OR “Genital disease*” OR “Sexually transmitted infection*” OR “Unsafe sex” OR “Sex education” OR “sexual literacy” OR “sexual health” OR “reproductive health” OR STI OR STD OR HIV
Concept 4: Help-seeking	“Health seeking behavio?r” OR “Help-seeking behavio?r” OR “Health system” OR “Health care” OR “Health service accessibility” OR “Health service” OR “Health information” OR “Health education” OR “Social support” OR “Primary health care” OR “HIV testing”

**Table 2:** General study characteristics, quality appraisal, and findings of fourteen studies addressing migrant sexual health help-seeking behavior in high income countries.

Title	Research Objective	Study Design	Conclusions/Recommendations
**Adedimeji et al. (2015) [36]** Increasing HIV testing among African immigrants in Ireland: Challenges and opportunities	To identify barriers for African migrants to access voluntary HIV testing, and to assess possible solutions to increase rates of HIV testing among this population.	**Setting:** Ireland **Inclusion criteria/eligibility:** Migrants from Africa, lived in Ireland more than 2 years and not previously diagnosed with HIV. **Sample:** 60 participants—focus groups (*n* = 56), interviews (*n* = 4). Mean of 4.7 years since migrating to Ireland. **Age range:** 18–64 years old **Gender:** Male and female **Type of study:** Qualitative; semi-structured interviews and focus groups **Recruitment:** Convenience and snowball sampling **Ethical approval:** Yes	**Conclusions:** Barriers to HIV testing in African migrants in Ireland were found, including fear of consequences of an HIV diagnosis (residency status and social relations) and test affordability. **Recommendations:** Involve stakeholders (immigrant group leaders, policy makers, health providers and religious leaders) in interventions to increase HIV testing to ensure cultural acceptability.
**Agu et al. (2016) [27]** Migrant sexual health help-seeking and experiences of stigmatization and discrimination in Perth, Western Australia: Exploring barriers and enablers	To explore barriers and enablers to sexual health help-seeking behaviors, and experiences of stigma and discrimination among migrants from sub-Saharan Africa and Southeast Asia living in Perth, Western Australia.	**Setting:** Australia **Inclusion criteria/eligibility:** Born in SEA or SSA, lived in Australia more than one year. **Sample:** 45 participants—21 from SSA, 19 SEA, 5 from other regions. 35 (76%) of participants had arrived in Australia less than 5 years. **Age range:** 18–50 years old **Gender:** Male and female **Type of study:** Qualitative; focus groups **Recruitment:** Purposive and snowball sampling techniques. **Ethical approval:** Yes	**Conclusions:** Barriers and enablers to sexual help-seeking behaviors included sociocultural and religious influence, financial constraints and knowledge dissemination to reduce stigma. Common experiences of stigma and discrimination (including in health care settings) and the social and self-isolation of people living with HIV. **Recommendations:** Address stigma and discrimination in health care settings. Provide culturally-appropriate sexual health knowledge that is group specific rather than targeted at migrants universally.
**Akerman et al. (2017) [35]** Healthcare-seeking behaviour in relation to sexual and reproductive health among Thai-born women in Sweden: a qualitative study	To explore sexual health help-seeking behaviors and views of HIV among Thai women living in Sweden.	**Setting:** Sweden **Inclusion criteria/eligibility:** Born in Thailand and living in Sweden less than five years **Sample:** 19 participants **Age range:** 24–50 years old **Gender:** Female **Type of study:** Qualitative; in-depth, semi-structured interviews **Recruitment:** Purposive sampling **Ethical approval:** Yes	**Conclusions:** Low sexual and reproductive health care use and low uptake of HIV testing. Women expressed low perception of risk to HIV. Barriers to healthcare included: language difficulties and low knowledge about the healthcare system. This resulted in a dependence on partners to access health services, or a preference to seek medical help in Thailand. **Recommendations:** Offer HIV testing as part of cervical cancer screening. Offer free health examinations to Thai migrants.
**Drummond et al. (2011) [29]** Barriers to accessing health care services for West African refugee women living in Western Australia	To examine barriers in accessing and utilizing health services of West African women refugees compared to Australian women.	**Setting:** Australia **Inclusion criteria/eligibility:** Refugee women from Liberia or Sierra Leone **Sample:** 51 women from Liberia or Sierra Leone and 100 Australian women (comparison). Women were newly arrived (less than 5 years) and had lived in refugee camps up to 10 years before resettlement. **Age range:** 20–67 years (West African), 18–90 years (Australian) **Gender:** Female **Type of study:** Quantitative; comparison study **Recruitment:** Snowball sampling **Ethical approval:** Yes	**Conclusions:** Barriers to accessing health care were negatively correlated with longer residence and higher education Emotional factors and service provider perceptions were major barriers to access healthcare services. **Recommendation:** Implement intensive health promotion campaigns through social networks and ethnic media.
**Dune et al. (2017) [28]** Culture Clash? Investigating constructions of sexual and reproductive health from the perspective of 1.5 generation migrants in Australia using Q methodology	To investigate the role of culture in constructions of sexual and reproductive health and health care seeking behavior from the perspective of 1.5 generation migrants	**Setting:** Australia **Inclusion criteria/eligibility:** Not described **Sample:** 42 participants with majority from SSA (43%) and SEA/EA (29%). Other regions included: Europe, Middle East and the Americans. 43% had arrived in the last 10 years. **Age range:** 18–39 years **Gender:** Male and female **Type of study:** Quantitative; Q methodology **Recruitment:** Purposive; flyers posted at relevant venues **Ethical approval:** Yes	**Conclusions:** Some migrants’ constructs of sexual and reproductive health changed when in a new culture; others had difficulty integrating new cultural values. Culture may be more easily adapted as many aspects of home (e.g., political, economically, etc.) do not exist in new country. Religion is portable, and may be the reason for an experience of ‘culture clash’ for some migrants.
**Guionnet et al. (2014) [37]** Immigrant women living with HIV in Spain: A qualitative approach to encourage medical follow-up	To examine the facilitators and barriers to medical follow-up among immigrant women living in Spain	**Setting:** Spain **Inclusion criteria/eligibility:** Women living with HIV; born in Spain, SSA or Latin America **Sample:** 26 participants—10 from SSA, 8 from Latin America, and 8 from Spain. **Age range:** 25–55 years old **Gender:** Female **Type of study:** Qualitative; semi-structured interviews **Recruitment:** Purposive sampling **Ethical approval:** Yes	**Conclusions:** Barriers for immigrant women living with HIV in continuing treatment included cultural, social, and gender roles, relationship with the healthcare system, and self-perception. **Recommendations:** Health professionals to work to identify and overcome barriers faced by patients in adhering to treatment
**Korner (2007) [32]** ‘If I Had My Residency I Wouldn’t Worry’: Negotiating Migration and HIV in Sydney, Australia	To describe the interrelationships between migration and resettlement, the Australian immigration system and living with HIV.	**Setting:** Australia **Inclusion criteria/eligibility:** People living with HIV, born in a non-English country, or speaking a language other than English at home **Sample:** 29 participants—16 (55%) in Asia, remainder from South America and Southern Europe; 11 (38%) were permanent residents, 12 (43%) had been in Australia longer than 10 years. **Age range:** 29 to 58 years **Gender:** Male and female **Type of study:** Qualitative; interviews **Recruitment:** Purposive sampling via a non-government organisation and a sexual health clinic **Ethical approval:** Yes	**Conclusions:** Main issue faced by migrants living with HIV was migration Uncertain immigration status can be a barrier to treatment, health care and support. **Recommendations:** Reduce barriers to accessing health services, including reviewing the practice of rejecting permanent residency applications of people living with HIV Address HIV-related stigma in migrant communities
**Lindkvist et al. (2015) [40]** Fogging the issue of HIV—Barriers for HIV testing in a migrated population from Ethiopia and Eritrea	To identify barriers faced by Eritrean and Ethiopian migrants in Stockholm, Sweden for HIV testing.	**Setting:** Sweden **Inclusion criteria/eligibility:** Born in Ethiopia or Eritrea **Sample:** 28 participants; focus groups (*n* = 21), interviews (*n* = 7). Arrival in Sweden ranged from 2 to 25 years. **Age range:** Age not reported **Gender:** Male and female **Type of study:** Qualitative; focus groups and interviews **Recruitment:** Purposive sampling **Ethical approval:** Yes	**Conclusions:** Main barrier was ‘fogging the issue of HIV’—categorised as hiding the truth, living in denial and seeking help outside the healthcare system. This was due to distrust of the healthcare system and fearing the consequences of living with HIV. **Recommendation:** Provide culturally appropriate information on HIV-related issues, in combination with offers of HIV testing early on arrival to Sweden.
**Manirankunda et al. (2009) [38]** “It’s better not to know”: Perceived barriers to HIV voluntary counselling and testing among sub-Saharan African migrants in Belgium	To examine the barriers, needs, and perceptions of HIV voluntary counselling and testing (VCT) among sub-Saharan African migrants in Belgium	**Setting:** Belgium **Inclusion criteria/eligibility:** Identified as SSA; English or French speaking. **Sample:** 70 participants. Mean duration of stay 8.5 years. **Age range:** 18–49 years **Gender:** Male and female **Type of study:** Qualitative; focus groups **Recruitment:** Purposive sampling **Ethical approval:** Yes	**Conclusions:** Multiple barriers to VCT identified including: fear of dying of AIDS, fear of stigma or discrimination and low perceived risk of acquisition. **Recommendation:** Implement VCT with pre- and post-test counselling, including via health services and via community outreach testing.
**McMichael and Gifford (2009) [30]** “It is Good to Know Now…Before it’s Too Late”: Promoting Sexual Health Literacy Amongst Resettled Young People With Refugee Backgrounds	To explore young refugees’ accessibility to health information	**Setting:** Australia **Inclusion criteria/eligibility:** From refugee background. **Sample:** 142 participants—interviews (*n* = 14), focus groups (n-128). Most participants were from Iraq, Afghanistan, Burma, Sudan, Liberia and the Horn of Africa. **Age range:** 16–25 years **Gender:** Male and female **Type of study:** Qualitative; focus group discussions and interviews **Recruitment:** Purposive sampling **Ethical approval:** Yes	**Conclusions:** Similar barriers were found to health service access as other young people Experiences of forced migration, displacement, and resettlement brings additional challenges. **Recommendation:** Improve accessibility of sexual health services to reduce poor sexual health outcomes and increase sexual health literacy.
**Rogers and Earnest (2014) [31]** A cross-generational study of contraception and reproductive health among Sudanese and Eritrean women in Brisbane, Australia	To assess knowledge and access to contraception and reproductive health of mothers and daughters from Sudanese and Eritrean backgrounds living in Brisbane	**Setting:** Australia **Inclusion criteria/eligibility:** Sudan or Eritrean women from refugee or migrant background **Sample:** 13 participants—8 aged between 35–55 years, 5 aged 18–30. **Age range:** 18–30 years, or 35–55 years **Gender:** Female **Type of study:** Qualitative; focus group discussions **Recruitment:** Purposive and snowball sampling **Ethical approval:** Yes	**Conclusions:** A range of barriers found to health service access and contraceptive use included: lack of cultural competency and ineffective communication by health care workers; poor knowledge of health care system and intergenerational culture clash in relation to sexual health education in the home. **Recommendations:** Provide sexual health information for new migrants during process of resettling Develop partnerships between health care professionals and CaLD communities Provide translated health information and access to interpreters Design culturally sensitive strategies for parents to communicate with their children about sexual health and enable parent-daughter transfer of health information.
**Shangase and Egbe (2014) [33]** Barriers to accessing HIV services for Black African communities in Cambridgeshire, the United Kingdom	To examine barriers faced by Black African communities to accessing HIV healthcare services.	**Setting:** United Kingdom **Inclusion criteria/eligibility:** From African communities. **Sample:** 30 participants; most aged in their twenties and thirties **Age range:** 21–65 years **Gender:** Male and female **Type of study:** Qualitative; focus group discussions **Recruitment:** Purposive sampling **Ethical approval:** Yes	**Conclusions:** A range of barriers found including language, limited knowledge of HIV, preference for traditional medicines and lack of cultural diversity among health service workers. **Recommendations:** Plan health services considering cultural diversity, including use of traditional medicine Ensure HIV workforce undertakes cultural competency training, and is culturally diverse.
**Thomas et al. (2010) [34]** “If I cannot access services, then there is no reason for me to test”: the impacts of health service charges on HIV testing and treatment amongst migrants in England	To examine the influence of England’s government health policy on migrants’ health seeking and HIV testing.	**Setting:** United Kingdom **Inclusion criteria/eligibility:** Living in the UK as a migrant **Sample:** 70 participants from South Africa, Zimbabwe, and Zambia **Age range:** Above 18 years **Gender:** Male and female **Type of study:** Qualitative; focus group discussions **Recruitment:** Purposive sampling **Ethical approval:** Yes	**Conclusions:** Changes in policy resulted in difficulties in accessing healthcare services due to cost and difficultly registering. **Recommendations:** Reverse the policy changes made Provide clear information and guidelines to both migrants and health workers in regards to accessing free health services.
**Yakubu et al. (2010) [39]** Sexual health information and uptake of sexual health services by African women in Scotland: A pilot study	To identify sources of sexual health information sought by African women in Scotland.	**Setting:** Scotland **Inclusion criteria/eligibility:** Women from Africa **Sample:** 96 survey respondents; 47% had lived in the UK less than 5 years. **Age range:** 16–55 years old **Gender:** Female **Type of study:** Quantitative; cross-sectional survey **Recruitment:** Purposive sampling **Ethical approval:** Yes	**Conclusions:** Poor knowledge of STIs and HIV and low uptake of sexual health services and regular screening. **Recommendation:** Develop collaboration between African communities in Scotland with the sexual health services to develop better HIV prevention program.
**Ussher et al. (2012) [26]** Purity, privacy and procreation: Constructions and experiences of sexual and reproductive health in Assyrian and Karen women living in Australia	To assess experiences of Karen and Assyrian woman refugees in utilizing SRH services in Australia	**Inclusion criteria/eligibility:** Women from Karen and Assyrian communities who arrived as refugees **Setting:** Australia **Sample:** 42 participants—28 (67%) from Karen communities. Karen participants had arrived on average 3.5 years ago. **Age range:** 25–45 years **Gender:** Female **Type of study:** Qualitative; focus group discussions **Recruitment:** Purposive sampling **Ethical approval:** Yes	**Conclusions:** Constructions and experiences of sexual health were closely tied to cultural, religious and gendered family views. **Recommendations:** Further research to explore interaction of gender, culture and migration process in the construction of sexual health.
